# Internal factors of entrepreneurial and business performance of small and medium enterprises (SMEs) in East Java, Indonesia

**DOI:** 10.1016/j.heliyon.2023.e21637

**Published:** 2023-10-30

**Authors:** Veronika Agustini Srimulyani, Yustinus Budi Hermanto, Sri Rustiyaningsih, Laurensius Anang Setiyo Waloyo

**Affiliations:** a Faculty of Business, Widya Mandala Surabaya Catholic University, Surabaya, Indonesia; b Management Study Program, Economic Faculty, Darma Cendika Catholic University, Surabaya, Indonesia; c Industrial Engineering Study Program, Engineering Faculty, Widya Mandala Surabaya Catholic University, Surabaya, Indonesia

**Keywords:** Business performance, Entrepreneurial internal factors, Innovative behavior

## Abstract

The analysis conducted in this empirical study is the direct and indirect impact of internal entrepreneurial factors on the performance of small and medium enterprises. These factors were identified from previous studies from various countries, such as entrepreneurial-innovative work behavior, entrepreneurial leadership, entrepreneurial self-efficacy, and entrepreneurial motivation, by taking the research object of small-medium enterprise owners in Java. East, Indonesia. Descriptive statistics and structural equation models were used to analyze the research data. Samples were taken with uncontrolled quota sampling techniques. The research data was collected by distributing questionnaires online with the Google form application and offline. The total respondents were 575 small-medium enterprise owners. The test results showed that internal factors such as entrepreneurial self-efficacy, motivation, and leadership could increase business performance significantly.In contrast, entrepreneurial-innovative work behavior cannot significantly increase business performance. Other test results showed that: 1) entrepreneurial self-efficacy significantly improved entrepreneurial motivation; 2) entrepreneurial leadership significantly improved innovative entrepreneurial behavior; 3) entrepreneurial motivation partially mediated the influence of entrepreneurial self-efficacy on business performance; 4) innovative entrepreneurial behavior did not mediate the influence of entrepreneurial leadership on business performance. The contribution of the results of this study provides additional empirical studies on the role of entrepreneurial leadership, which significantly increases innovative entrepreneurial behavior. There is a lot of potential to develop business existence through entrepreneurial leadership through innovative behavior in entrepreneurship. Another contribution of this study is that entrepreneurial self-efficacy is a dominant predictor of business performance in SMEs and significantly increases entrepreneurial motivation. These findings reveal the importance of self-efficacy for SMEs because high self-efficacy for business actors is expected to increase entrepreneurial self-efficacy and teamwork motivation in helping to achieve business performance.

## Summary highlights

• Contributions, This paper contributes to developing research on internal entrepreneurial factors (entrepreneurial self-efficacy, entrepreneurial motivation, and entrepreneurial leadership) that affect the resilience and business performance of SMEs in East Java during the Covid-19 pandemic. This study also shows entrepreneurial leadership's positive and significant effect on innovative entrepreneurial behavior in SME owners in East Java.
• *Research questions and purpose*: This study focuses on internal entrepreneurial factors to analyze the direct and indirect impact of internal factors that may affect the business performance of SME owners in difficult times such as the Covid-19 pandemic.
• *Primary research methodology and information:* Descriptive statistical analysis and SEM path analysis with the Sobel test were used for the quantitative study.
• *Database/Information*: Data collection was carried out by distributing questionnaires. The questionnaires were distributed online with the google form application through social media and offline by visiting directly at the business location.
• *Results/findings*: The test showed that entrepreneurial self-efficacy, motivation, and leadership significantly improved business performance, while innovative entrepreneurial behavior had no significant impact on business performance. Other test results showed that: 1) entrepreneurial self-efficacy significantly improved entrepreneurial motivation 2) entrepreneurial leadership significantly improved innovative entrepreneurial behavior; 3) entrepreneurial motivation partially mediated the influence of entrepreneurial self-efficacy on business performance; 4) The innovative entrepreneurial behavior did not mediate the influence of entrepreneurial leadership on business performance.
• *Limitations and future research directions*: The samples were obtained based on the uncontrolled quota sampling technique, which is a sampling technique that is always carried out by not imposing any restrictions on the choice of researcher samples because the population of SMEs in East Java is substantial and continues to grow. There is no adequate SME database in every city and district in the East Java region. Therefore, the results of this study cannot be generalized to SMEs throughout East Java, Indonesia. The results of this study have also not been able to prove the significant impact of innovative entrepreneurial behavior on business performance. Subsequent research developments can be retested using similar research objects (SMEs) but focus on creative industries. Research field exploration needs to be conducted. Apart from exploring the determinants of business performance, external entrepreneurial factors such as digital marketing, human technology, and financial technology can be considered.
• *Practical implications and recommendations*: 1) Entrepreneurial motivation for SMEs needs to be increased to foster innovative behavior of business actors and work teams so that creativity and business innovation can be optimally increased; 2) Entrepreneurial leadership for SMEs needs to be improved because entrepreneurial leadership plays an important role in the survival, success, and growth of businesses through creativity and innovation; 3) There is a need to continue to increase entrepreneurial self-efficacy for business actors because, with high entrepreneurial self-efficacy for SMEs, it is hoped that this will increase their sense of self-efficacy and motivation of their work team; 4) The government needs to increase its intervention in the process of resolving systemic market failures and be more proactive in supporting innovative business activities.

## 1 Introduction

One of the pillars of the economy in Indonesia that plays a role in economic growth is SMEs. SMEs are highly heterogeneous economic units with varying degrees of complexity (International Labour Organization (ILO), 2021). The ILO report in August 2019 revealed statistics from 99 countries showing that 70 % of job opportunities are generated by micro and small businesses and entrepreneurs [1]. SMEs in Indonesia play an important role in economic growth and job creation. This was proven in 2017; SMEs accounted for 97.3% of all businesses in Indonesia, contributed 37% of the country's GDP, and provided 66% of total employment [2]. Micro, Small, and Medium Enterprises (MSMEs) thrive in some areas. This can be seen from the data reported by the Ministry of Cooperatives and Small and Medium Enterprises. The total number of MSMEs in Indonesia will reach 8.71 million business units in 2022, and the island of Java dominates this sector. Half of Indonesia's population lives on Java Island, and Java is Indonesia's largest economic center [3]. East Java Province is the third province in Indonesia, making a substantial contribution to the country's economy [4]. SMEs in East Java Province are the second largest contributor to the national economy after DKI Jakarta. For example, in Malang City, East Java, the existence of SMEs contributes to restoring the economy, especially those active in the online market.

The development of SMEs in various countries has experienced a lot of progress, including in Indonesia. SMEs are growing rapidly in Indonesia, especially businesses that produce Indonesian specialties [5], but during the COVID-19 pandemic, culinary businesses in Indonesia also experienced a decline in sales. The COVID-19 outbreak has negative and positive sides; for example, someone can think creatively to keep balancing income to maintain the economy [6]. Sales turnover in several SMEs during the Covid-19 pandemic had experienced a significant decline, but online sales facilities or digital technology helped SMEs get consumers, so the business continued to run. The number of new SMEs in DKI Jakarta increased during the COVID-19 pandemic; 200,000 new entrepreneurs were functioning, and 289,370 were registered and promoted, supported by the DKI Jakarta Provincial Government [2]. [1] conducted a study on the relatively fast recovery of SMEs during the pandemic in Beijing, China, revealed that the direct impact of the pandemic on the market was the most prominent, and government policies could indirectly significantly reduce the negative impact of the pandemic on SMEs. In a transitional economy, the competitive business environment has made it difficult for high-tech entrepreneurship-based SMEs to survive and grow [7].

During a pandemic, creative behavior is very important, especially for entrepreneurs. Entrepreneurial creativity in adjusting business models according to current conditions plays an important role in business sustainability. One of the business models during a pandemic that can be adopted by entrepreneurs, including entrepreneurs in the culinary field, is to use online transactions. It is shown that, during the pandemic, there was an increase in goods delivery services, especially from e-commerce, necessities services, and the demand for vegetables. Delivery services such as GoFood and GoSend are the savior of SME businesses in Indonesia during the pandemic [8]. analyzing the performance of SMEs in Indonesia revealed that entrepreneurial competence significantly moderates the relationship between e-commerce and SME performance. Furthermore [9], explored the impact of reduced Covid-19 performance on SMEs in Guangdong Science and Technology Park in China and discussed the management innovation response and corporate organizational resilience mechanisms based on SME questionnaire survey data, finding the results that criticality and the disruption of the Covid-19 pandemic have not only caused a decrease in the performance of SMEs but has also enabled SMEs to carry out management innovations that indirectly reduce performance declines by encouraging organizational resilience.

A study from Ref. [10] revealed that young East Java entrepreneurs have a slightly higher self-efficacy score in the financial sector compared to self-efficacy related to soft skills. With strong self-efficacy, SME actors will not fear facing challenges and overcoming obstacles and can make better business decisions. During the Covid-19 pandemic, SMEs in East Java were forced to change the direction of their business strategy; some even experienced a crisis [11]. SME owners have tried various strategies to survive and earn income during the Covid-19 pandemic. Starting from efforts to switch to other businesses that are felt to provide better opportunities, making cost savings, seeking loans, negotiating with suppliers, trying to take advantage of technology and selling online, or innovating products that can meet changing market needs due to the pandemic.

Entrepreneurial behavior (EB) in the behavioral approach is understood as an interactional process, which is a product of individual, organizational, and environmental influences [12]. EB is based on specific values or beliefs and needs that provide individuals with intrinsic motivation and self-determination to engage in entrepreneurial behavior [13]. The main individual characteristics that shape entrepreneurial decisions are personality traits, motivation for growth, individual competencies, and personal background [14]. Individual factors determining business success include entrepreneurial culture, personality traits, knowledge and experience, and growth aspirations [15]. Entrepreneurial experience, managerial skills, and education positively impact business performance, especially when accompanied by growth motivation [16]. EB can encourage entrepreneurs to take action on business startup opportunities [17]. EB has an impact on business performance (BP) [15,18].

EB is one of the capital entrepreneurs, and business managers can use it to win the business competition. EB is essential in improving social and hard skills [19]. Examples of EB are personality characteristics that can be seen and observed, such as entrepreneurial creativity, entrepreneurial discipline, level of entrepreneurial self-confidence, level of courage to take business risks, level of self-motivation, and leadership practice. EB is an internal factor of entrepreneurship. Internal factors are essential in determining the comparative advantages of the business [20]. Entrepreneurial self-confidence and persistence in managing business units (self-efficacy), entrepreneurial personality, entrepreneurial courage in taking risks, and entrepreneurial motivation (EM) are crucial for achieving business goals [21]. EM is an important corporate capability resource for spreading sustainable competitive advantage [22]. Meanwhile, the problems often faced by SMEs in Indonesia to develop are more influenced by internal factors [23].

Research [20] conducted on SMEs in five provinces in Indonesia, namely East Java, West Sumatra, North Sumatra, West Nusa Tenggara, and East Nusa Tenggara, revealed that internal factors (RBV), external (Market Based-View), and management risk has a significant positive effect on the performance of SMEs. Empirical studies on SMEs in East Java showed that an increase in sales, profits, assets, and job creation could significantly improve the performance of SMEs [4]. Research on SMEs in Padang City [23] showed that business knowledge, business skills, and innovation significantly affected BP. The study's results on the food and beverage business in Bangkok showed that several essential variables influence the business's success: Human Resources Management (HRM), management strategy, innovation, technology, product quality, leadership, team potential, and branding [24]. Furthermore [25], examines the importance of SMEs in socio-economic development in Kosovo and shows that internal SME factors, such as managerial skills and competencies, business experience, access to finance, and technological capabilities, significantly impact SME performance.

Entrepreneurial persistence is one of the EB, an individual characteristic of entrepreneurs in the form of personality traits and human capital [26]. The character of entrepreneurship, learning, and competence have been identified as the most important entrepreneurial factors that determine SMEs' performance [4]. Pioneers of a business generally have higher self-efficacy so that they have better abilities in their roles and duties to become entrepreneurs, such as innovation in product marketing, business management, courage to take risks, and the ability to manage finances than individuals who manage businesses who are not business pioneers [27]. Entrepreneurial self-efficacy (ESE) can be seen in individual behavior as entrepreneurs and shown through confidence in entrepreneurial activities [13]. ESE can significantly increase EM and the business success of SMEs [28]. Several other studies show that ESE can significantly improve entrepreneurial behavior pioneers [27]; ESE also influences a business's success by improving individuals' performance in various fields, including entrepreneurship. ESE also influences a business's success by improving individuals' performance in various areas, including entrepreneurship [[29], [30], [31]]. ESE influences EM [32,33] and entrepreneurial, innovative behavior [34].

Other empirical studies show that BP is positively and significantly affected by entrepreneurial leadership (EL) [35] and innovative behavior (IB) [36]. Other studies showed that EL affects IB [37,38]. During the Covid-19 pandemic, all economic sectors, especially SMEs, needed creativity in innovation. To innovate also requires motivation and creativity in a person to help the open mind of SME owners in making a change to continue to survive and succeed in their business [39]. [29] analyzed the financial performance of SMEs in Surabaya, showing that innovation, courage to take risks, and proactive entrepreneurship can improve financial performance. During the pandemic, SMEs must be more innovative in promoting and selling online and sensitive to the community's needs due to Covid-19.

This research focuses on EB, an internal factor of entrepreneurship, to analyze EB's direct and indirect influence on SMEs. These factors were identified from previous studies, namely ESE, EM, EL, and EIB, by taking SME objects from various districts and cities in East Java.

### 1.1 Literature review

The company's success is determined by internal resources and the important influence of company resources on competitive position [30]. SMEs must reconfigure, reallocate, and recombine their resources to achieve their goals [14]. Based on a review of various kinds of literature that classifies factors related to corporate-level performance, they can be grouped into three, namely: 1) individual factors (related to the employer or entrepreneur); 2) organizational factors (company and strategy and its own characteristics); 3) environmental factors (industrial and regional/national environment where the company develops its activities) [15]. If a company wants to improve business performance, several efforts that must be made are increasing its entrepreneurial competence and adopting e-commerce [8]. The Resource-based view (RBV) explains that several competitive advantages come from aligning skills, motivation, etc., with organizational systems, structures, and processes to achieve organizational capabilities [31]. In RBV, the focus of attention is on internal factors, which are the fundamental variables of the company and its performance [20]. RBV can be grouped into three resources, namely: (1) tangible resources such as physical buildings, (2) intangible resources such as knowledge or motivation, and (3) capabilities [22].

Entrepreneurship is a country's development potential in terms of the number and quality of entrepreneurs. Entrepreneurs and company managers can carry out enterprise development by managing company assets to influence their company's dynamic capabilities [33] positively. Dynamic capabilities are a positive function of the corresponding dynamic capabilities of individual and collective organizational roles [33]. Wilkens et al. [40] reveal four categories of micro-foundations of dynamic capabilities: (1) leadership behavior, (2) team interaction, (3) individual capabilities, and (4) job characteristics. Studies related to managers (entrepreneurial) in organizations focus on executive leadership as a driver of dynamic capabilities [41]. The theory that illustrates how the behavior of a manager, his cognition, and all the environments operate as determinants of interaction with one another is the social cognitive theory (SCT) [42]. For example, a manager's ability to perceive, seize and reconfigure [33] corporate assets (behavior) works in a context that accepts a willingness to seek new opportunities (environment), as well as individual characteristics such as self-efficacy, self-motivation, and expertise (personal factor). Manager competence consists of two main dimensions: 1) manager behavior and 2) aspects: nature, social roles, and skills of managers [18].

### 1.2 Business performance (BP)

In business competition, BP is most important for business success. BP can be measured through four perspectives: financial, customer, innovation and learning, and internal processes [43]. According to Ref. [44], BP is a strategic aspect of business management. It is an integrated part of all business operations carried out by business managers in an effort by managers to grow the business they run. Sanchez & Marin [45] assessed the performance of SMEs through three aspects: 1) profitability looks at business performance from the aspect of achieving financial goals that the company has designed; 2) productivity, looking at the achievement of business performance in fulfilling customer wants and needs, as well as employee productivity; 3) the market looks at business performance in terms of product sales achievement, market position, and market share. BP significantly affects business development. In general, a company's BP consists of two groups, namely financial performance (a performance that monetary units and financial activities can measure) and non-financial performance (a performance that cannot be measured with monetary units, such as the quality of brand strength and reputation, customer satisfaction with products, organizational performance, and innovation performance) [46]. BP can be seen from the level of sales, return on capital, turnover rate, and market share that has been owned [47].

BP measurement needs to be carried out by entrepreneurs and business managers in order to see the success of the strategies and capabilities implemented [48]. BP can be interpreted broadly and generally and has a complex construction. Various studies measure BP in financial aspects, and several others consider business success in non-financial performance indicators. A measure of business performance from the financial aspect describes the achievement of an entrepreneurial business measured by the value of money and financial operations. BP, seen from non-financial factors, is a business achievement that cannot be measured in value by money, for example, brand reputation, customer satisfaction, and business innovation activities [49]. Many factors impact the performance of SMEs, both internally and externally. The entrepreneurs' internal factors include confidence and motivation [50].

The performance of SMEs will depend on the business segments that have been successfully achieved. Some obstacles to achieving success that entrepreneurs need to pay attention: poor quality EL, lack of planning, lack of motivation, fewer human resources, low entrepreneurial competence, high production costs, limited market access, low ICT skills, and limited capital [4]. The success and sustainability of SMEs cannot be separated from the internal factors of business owners. The existence of self-confidence in the abilities possessed, called ESE, can increase self-motivation in achieving business performance targets. The strong desire of business owners to develop a business can give rise to a strong motivation to run a business successfully, which is an ideal that business owners expect. Entrepreneurs with strong self-motivation in running a business will desire to achieve business success. ESE and EM are components of entrepreneurial values that enhance BP. EM has a significant role to play in the progress of SMEs.

### 1.3 Entrepreneurial self-efficacy (ESE)

ESE is one of the internal factors of entrepreneurship, namely confidence [50], which is described as the level of self-confidence and entrepreneurial spirit in the competence in carrying out the role of entrepreneurship. ESE is the intensity of an entrepreneur's confidence about whether the skills they possess can complete various entrepreneurial activities, which illustrates the belief that entrepreneurs are equipped with adequate competencies to influence their environment and succeed in entrepreneurial roles [51,52]. Self-efficacy (SE) in domain-specific constructs, such as ESE, has theoretical roots in the agencies' perspective in which individuals demonstrate interaction reciprocally with the internal and external environment [53]. SE is the belief in a person's ability to succeed and achieve a certain level of performance [51]. SE is the belief in the ability to organize and give rise to the behaviors needed to produce specific skills. Entrepreneurs with high SE can change business productivity through their competence, skills, and entrepreneurial knowledge [52]. ESE is one of the personal attributes defined as a belief in a person's ability to do different things on entrepreneurship-related tasks [54]. ESE is the hope of entrepreneurial success based on the belief in self-ability that can impact the business environment so that entrepreneurs can control actions to obtain the desired results [22]. ESE can be interpreted as a person's trust and confidence in one's ability to achieve various entrepreneurial tasks. ESE turns entrepreneurs' confidence into efforts, which improves the company's performance [55], improves the firm's innovation behavior [56], and increases entrepreneurial innovation behavior [34].

### 1.4 Entrepreneurial motivational (EM)

Motivation is an internal factor of entrepreneurship that can impact BP. Entrepreneurial motivation combines the psychological attributes of individual entrepreneurs and the environment that stimulates entrepreneurial actions, such as exploring market opportunities, product innovation or development, establishment, management, expansion, and business diversification, and business performance [57]. Entrepreneurial motivation is the main aspect of pushing someone to start a business [58]. The motivational factor becomes an important internal factor among entrepreneurs to develop business performance; motivation significantly affects performance [59]. EM is an asset, especially for entrepreneurs involved in MSEs [60]. The motivational factor is an important factor that encourages a person to engage in business activities.

In contrast, the ability factor is used so that a person has sufficient ability to carry out these activities [61]. Motivational factors influence the entrepreneur's business decision [62]. EM is considered a resource of enterprise capabilities, an important resource for creating a sustainable competitive advantage [22]. EM includes motivations directed toward achieving entrepreneurial goals. In narrower terms, expectancy theory reveals that specific and periodic information regarding entrepreneurial opportunities can increase an individual's expectations that entrepreneurial efforts will deliver results, thereby increasing EM. Global Entrepreneurship Monitor (GEM) distinguishes entrepreneurial motivation into two different types, namely motivation, which is interpreted into entrepreneurial needs and opportunities [63].

### 1.5 Entrepreneurial leadership (EL)

Mental attitude is one of the internal factors of entrepreneurship that can increase BP (Putra et al., 2019), where leadership is part of the managerial role related to the entrepreneurial mental attitude in leading organizational resources to achieve the desired performance. The study of the impact of entrepreneurial leadership factors on business performance, especially for SMEs, is more about how SME leaders view managed businesses as a 'platform' for their own business. The EL theory states that based on the functional abilities of the leader, entrepreneurial leadership (like neo-charismatic and value-based leadership) encourages group members to discard conventional tasks and directs group members' energies toward implementing innovative and entrepreneurial actions [64]. Entrepreneurial leadership is called a transformational leadership style because it builds the regeneration of leaders by instilling creativity, motivation, and the ability to take risks [65]. Green transformational leadership improves environmental performance in SMEs [66]. Entrepreneurial leadership has three common factors: proactivity, innovation, and risk-taking [64,67]. Successful leaders and entrepreneurs have something in common in risk-taking and creativity, but the role of an entrepreneur is more complex than that of a leader [37]. Successful entrepreneurs run their businesses with a strong leadership commitment, which helps entrepreneurs maintain future business success [68]. EL is not a position but a process [69]. EL arises at crossing entrepreneurship and leadership [49]. Based on a separate literature study on EL, several characteristics are common to leaders and entrepreneurs: vision, problem-solving, decision-making, risk-taking, and strategic initiatives [70]*.* Several components identify EL: innovativeness, proactiveness, risk-taking, organizing, and conducting [71]*.* EL is related to how to build long-term mutual relationships along the organization's value chain, where effectiveness is determined by the leader's ability to influence others, set direction, communicate, motivate, develop change, handle resources strategically, and encourage others to act profitably and seek opportunities competitively [72].

### 1.6 Entrepreneurial innovative behavior (EIB)

Innovation behavior (IB) for entrepreneurship or EIB is the key to addressing dynamic changes in the external environment for continuity and is a driving force for companies to be able to gain a competitive advantage [34,73]. IB is an internal entrepreneurial factor that can improve BP, especially related to technical ability factors. IB is involved in the innovation process as the beginning of a creative result [38]. IB is a construct that captures all the behaviors in which employees can contribute to the innovation process [74]. Innovative behavior is not only related to creativity, but this innovative behavior is more complex when compared to creativity because innovative behavior reaches the stage of applying the ideas that have been created. IB can be defined as an individual who practices innovation by creating and applying new ideas in the workplace [37]. IB as a reflection of creating something new or different. Innovation behavior is the tendency of innovation to be innovative or adaptive [75]. IB is individual behavior directed at the intentional initiation and recognition (in a work role, group, or organization) of a new and beneficial idea, process, product, or procedure [76].

There are four dimensions of EIB as follows: a) Opportunity exploration, the process of innovation is determined by the opportunity by which this opportunity will trigger individuals to find ways to improve services and delivery processes or to try to think of a new alternative to the work process, product or service; b) Idea generation is the re-management of existing information and concepts to improve performance; c) Championing, involves behaviors to seek support and build coalitions, such as engaging and influencing employees or management, and negotiating on a solution; d) Application, individuals not only think about creative ideas on something but also evaluate and apply the idea to real action [77].

## 2 Hypotheses

### 2.1 Effect of ESE on BP

Someone with strong self-efficacy for entrepreneurship usually, without hesitation, takes well-calculated risks for business progress so that it can impact business success. The higher the level of SE owned by an entrepreneur, the higher the level of self-success related to the entrepreneur's tasks will be [22]. Confidence, as one aspect of ESE, will make one feel optimistic about carrying out the role of an entrepreneur. The results of previous empirical studies showed that ESE positively affected business performance significantly [75] and business success [28]. With reasonable confidence and ability, entrepreneurs will plan well in running a business and impact business performance. ESE can influence business performance through attitudes and behaviors through entrepreneurial actions oriented towards achieving achievement or performance by utilizing their abilities. With a strong ESE, SME owners will not be afraid to face challenges, overcome obstacles, and be able to make better business decisions. Based on these findings, we stated our first research hypothesis.

H1 ESE significantly improves BP.

### 2.2 Effect of ESE on EM

EM includes motivations directed toward achieving entrepreneurial goals. In narrower terms, expectancy theory reveals that specific and periodic information regarding entrepreneurial opportunities can increase an individual's expectations that entrepreneurial efforts will deliver results, thereby increasing EM. Global Entrepreneurship Monitor (GEM) distinguishes EM into two types: motivation, which is interpreted into entrepreneurial needs and opportunities [78]. ESE can keep the EM going for a long time because only if the entrepreneur has a strong belief in entrepreneurial success and confidence in entrepreneurial success will the entrepreneur be motivated to run the business. The existence of confidence in the ability possessed can increase self-motivation in achieving targeted business success. Some empirical studies show that the stronger the ESE owned by entrepreneurs, the stronger the EM owned by entrepreneurs [39,33,75]. Based on these findings, we stated our second research hypothesis.

H2 ESE significantly improves EM.

### 2.3 Effect of EM on BP

Motivation is the inner drive that ignites and maintains behavior to make ends meet. EM plays an important role in business performance [79]. EM is the driving motive in a person's heart to do or achieve a particular business goal. Strong-motivation entrepreneurs will make positive choices in satisfying self-desires [22]. EM can be defined as a set of motives, such as a high need for achievement, a moderate need for power, and a low affiliate motive, which encourages people to set up and run their own companies. Higher EM will lead entrepreneurs to higher efforts to pursue achievements on BP. The results of a study on SMEs in Bandung [22] showed that EM measured by achievement, risk propensity, and self-efficacy positively and significantly affected company performance. Business success depends on the entrepreneur's level of motivation [5,28]. EM, directly and indirectly, improves business performance with strategic leadership as a mediator [79]. EM impacts increasing business segments that can be assessed by increasing business scale, income, competitive advantage, work planning, communication, and cooperation [59]. Another study [80] showed that four motivational factors of entrepreneurship (independence, need for achievement, social recognition, and financial reward) positively and significantly influence the survival success of a business. The results of an empirical study on small business owners in nine districts of Baghdad, Iraq, show that EM significantly positively affects BP and motivates entrepreneurs to be innovative and creative in their business activities [81]. Based on these findings, we state our third research hypothesis.

H3 EM significantly improves BP.

### 2.4 Effects of EL on BP

Managers in each organization play the role of leaders, where the leader in each organization plays the most critical role in obtaining organizational success [24]. SME leaders can use policies to influence the company's strategy and performance significantly. EL style positively influence organizational performance [69] and entrepreneurial success [68]. Another empirical study [82] proved that the EL behavior of SME managers or owners as measured from four constructs (miner behavior, explorer behavior, accelerator behavior, and integrator behavior) affected the performance of these SMEs significantly. Entrepreneurs who have a leadership spirit can have high business performance. Entrepreneurs who can be innovative and persistent can create better business performance. This is evidenced in an empirical study on 137 entrepreneurs, which showed that EL significantly positively influenced BP in the creative economy sector in Malang Raya [83]. Based on these findings, we state our fourth research hypothesis.

H4 EL significantly improves BP.

### 2.5 Effects of EL on EIB

Innovation is the company's key factor in achieving long-term sustainability success and competitive advantage, indirectly influenced by entrepreneurship leadership with innovative work behavior [84]. The relationship between EL and innovative behavior is based on social cognitive theory [85] and a RBV of the firm [86] in explaining how entrepreneurial leaders increase the capacity of firms to generate innovation and sustainable competitive advantage through increased motivation and competence of individuals and teams work to generate and implement new ideas. Entrepreneurs must develop new leadership skills by stimulating innovation behaviors and leading the innovation process in the business of their leads. Entrepreneurial leadership can help companies create valuable innovations by engaging people as resources, knowledge, and ideas. In previous empirical studies, IB was significantly positively influenced by EL [5,43,83,84]. EL has much in common with transformational leadership (TL) in that the leader generates higher performance by appealing to the higher needs of followers [64]. Al-Omari et al. state that transformational leaders improve the supporting actors of innovative work behavior and stimulate employees' work behavior to commit to their efforts for the betterment of the organization [87]. The study by Nasution and Syahrizal [88] showed that inclusive leadership has a significant and positive effect on IWB in the workforce of SMEs in Muara Bulian. The results of empirical studies by Mehmood et al. [89] show that EL, directly and indirectly, impacts innovative behavior through psychological empowerment. Another study [90] showed that the influence of EL affects the capacity of innovation, and EL and the capacity of innovation affect the competitive advantage of micro-enterprises in Bangka Regency. This means EL is an important aspect of ESE that can improve EIB because EL provides greater opportunities for employees to develop employee creativity. Based on these findings, we state our fifth research hypothesis.

H5 EL significantly improves EIB.

### 2.6 Effects of EIB on BP

BP cannot be separated from IEB [91]. An entrepreneur who adopts and integrates the IB philosophy automatically improves aspects of the work environment whenever there is an opportunity to perform, and in general, the entrepreneur is willing to adopt improvements proposed by colleagues or others. EIB can define innovative capabilities, including technological innovation capabilities that can ultimately positively impact BP [49]. The results of another study [92] conducted to examine the influence of EIB on the performance of SMEs in the service sector in Sabah, Malaysia, showed that EIB had a positive and significant effect on organizational performance. Another study [36] showed a significant positive influence of EIB on BP. EIB causes changes in organizational performance [91]. This indicates that the increase in innovative behavior in managers and employees in a company can make work more efficient and effective to improve BP. Based on these findings, we state our sixth research hypothesis.

H6 EIB significantly improves BP.

### 2.7 Effect of ESE on BP through EM

ESE generally refers to social cognitive theory and planned behavior theory, which can aid people's understanding of why individuals with higher levels of ESE achieve greater motivation, behavior, persistence, and entrepreneurial performance and how ESE enables individuals to deal with situations that do not sure and challenging at work [53]. ESE is a strong belief in an individual's ability to successfully perform roles and jobs as an entrepreneur. ESE is needed so that EM grows strongly because EM factors can influence a person's behavior in their entrepreneurial role. The higher an entrepreneur's self-efficacy, the higher the motivation for entrepreneurship, thus increasing business success. The stronger the EM, the more business performance will increase. The results of previous empirical studies [28] show that ESE indirectly influences business success in culinary SMEs in East Java through EM as a mediator. The higher the ESE that a person has, the higher the EM, so business success will also increase; on the contrary, the lower the ESE that a person has, the EM will decrease so that BP will decrease. Thus it can be concluded that ESE affects BP through EM as a mediator. Based on these findings, we state our seventh research hypothesis.

H7 ESE affects on BP through EM as a mediator.

### 2.8 The effect of EL on BP through EIB

EL is seen as effectively increasing competitiveness in overcoming uncertain environments and achieving sustainable organizational development, so this leadership model fosters innovative work behaviors. With IB, it is expected to generate new and valuable ideas, get sponsors, implement them, and be promoted [92], which is expected to improve business performance. The involvement of individuals in entrepreneurship is closely related to innovation behavior. With the Covid-19 pandemic, business owners are expected to be able to find innovative ways to maintain the business that has been run so that it does not experience bankruptcy. To face the current situation, IT encourages SME owners to play a greater role in creativity and innovative behavior through various things such as information technology, transportation facilities, and others. As an innovative effort is considered a source of economic competitiveness, SMEs and innovations must synergize and integrate. Previous empirical studies [7] showed the influence of innovative environmental mediation on the relationship between EL and EIB and the role of ESE moderation in the relationship between EL and EIB in technology-based SMEs. Meanwhile, a study [93] revealed that team creativity, dynamic capabilities, and competitive advantage fully mediate the effect of EL on BP.

H8 EIB affects BP through EL as a mediator.

**Fig. 1:**
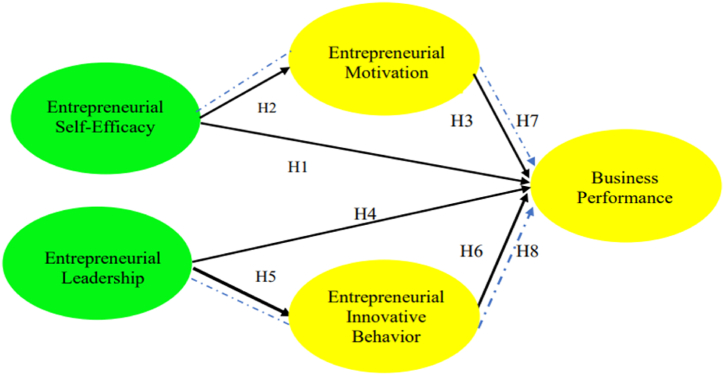
Theoretical framework.

Based on empirical studies and a review of the literature in the previous description, an entrepreneur's business performance (BP) is influenced by various factors originating from the entrepreneur himself, including entrepreneurial self-efficacy (ESE), entrepreneurial motivation (EM), entrepreneurial leadership (EL) and entrepreneurial innovation behavior (EIB). Based on previous empirical studies, it was also found that there is a mediating role of EM in the influence of ESE on BP and a mediating role of EIB in the influence of EL on BP. The theoretical model in this study is presented in Fig. 1.

## 3 Methods

### 3.1 Data collection

Data collection techniques in this study were very limited due to the Covid-19 pandemic situation, so data collection was carried out in person; researchers considered geographical proximity aspects, while data collection was carried out online through representatives of SMEs who are members of the "Citra Tangguh Harapan" Community, totaling 369 actors. Business. Because the size of the SMEs population in East Java is not known with certainty, the sampling technique used is uncontrolled quota sampling, which is carried out without restrictions on selecting research samples, meaning that within a predetermined sampling period, the researcher determines a certain amount. Limitations in obtaining samples: Data collection was carried out in two ways, namely online using the Google form (distributed through the WA Group) and offline channeled directly by involving research team partners located in creative industry centers and food and beverage industries located in Madiun City, Madiun Regency, Magetan Regency, Sidoarjo Regency, and Malang Regency. This research questionnaire can be seen in the supplementary material. Measuring all research variables using a 5-point Likert scale.

The research method uses descriptive statistical analysis with SPSS, Structural Equation Model (SEM) with Lisrel software and mediating analysis with the Sobel test online. SEM is a regression-based approach to test original research models with multiple constructs and measures [94]. SEM is used to test research hypotheses, as previous studies have done for quantification and theory testing, especially in management [95]. Analysis of the measurement model, using a two-step approach: 1) analysis of the measurement model with Goodness of Fit Indices, validity, and reliability tests; 2) If the three tests state good fit, proceed with the structural model analysis. Sobel tests online (calculation for the Sobel test) (http://www.quantpsy.org/sobel/sobel.htm) were conducted to test the strength of the indirect effect of the independent variable on the dependent variable through the mediating variable [93,94].

### 3.2 Operational definition and variable measurements

**ESE** is operationally defined as the self-confidence an individual possesses in performing various roles of a particular task in entrepreneurship. The ESE measurement refers to the opinion of [51], which consists of three main dimensions of ESE assessment: 1) individual beliefs (the belief of business actors in overcoming obstacles in doing business, developing its current business, optimistic in obtaining adequate profits); 2) actions (business actors: can compete with similar business actors; can manage its resources properly; can work well; tireless in facing challenges; can motivate employees to continue to work hard); 3)cognitive resources (business actors: can accept the consequences in running their business; can accept the uncertainty of income).

**EM** is operationally defined as a condition from within internally that encourages individuals consciously to carry out activities to achieve the desired goal of doing business. Measurement EM refers to previous studies [17,77] which included aspects of 1) financial reason (business actors get income every month; can improve the family economy; can use the capital owned to develop the business); 2) social reason (business actors are more appreciated in the social environment; want to be known by more people; want to be a role model for others; feel more honored than working in other people's business); 3) service reason (business actors can open new job opportunities; can meet market demand); 4) self-fulfillment reason (business actors can have their own business; being able to divide my time between business and family as opposed to working in someone else's company; can start their own business; does not depend on the business owned by the family/parents).

**EL** is operationally defined as an act of change that leads to increased creativity, innovation, intuition, and the ability to lead, motivate, and dare to take risks in the organization of the business. The measurement of EL refers to Ref. [4], which includes aspects: 1) the ability to motivate (business actors can increase employee morale; can provide direction to employees to work better); 2) achievement-oriented (business actors: can pay more attention to the business or effort being carried out; can delegate tasks properly; can oversee the process of activities in the company from upstream to downstream; willing to change plans that have been planned if there is input better); 3) creative (business actors: actively seeking new ideas for new products or business processes; can see opportunities in the market; can persuade others to think about creating a new product or business); 4) flexible (business actors: can provide a positive response to the changes that occur; can respond quickly to changes that occur); 5) persistent (business actors: can handle pressure at work; can take real action; can overcome any obstacle, no matter how difficult it is; continue to carry out their work even though it is full of challenges); 6) risk-taking (business actors: willing to bear the possibility of the company's financial losses, willing to bear the possibility of losses in the social aspects of life); 7) visionary (business actors: have ideas about future business; can notify employees about business developments undertaken; can convey plans and expectations of business actors for the future business they have to employees; can convince employees about the prospects of the business they have).

**EIB** is operationally defined as behavior related to creativity up to the stage of applying the ideas that have been created. EIB measurement refers to previous research [76] using four dimensions: 1) opportunity exploration, dealing with opportunity discovery as a preliminary process of innovation; 2) idea generation, dealing with products, services or processes, new markets, process improvements, and problem identification; 3) championing, namely the promotion of ideas, dealing with fighting for ideas to be relevant for implementation, and 4) application, namely the realization of the implementation of ideas, dealing with the behavior of realizing ideas.

**BP** is operationally defined as the result of work achieved as a whole that generally includes two components: financial performance and non-financial performance. Business performance is measured through subjective measurements and self-reports by owners/managers, which have been used consistently in previous studies [95,96]. 10.13039/100004364BP measurement indicators from the point of view of business actors refer to previous studies [29,31], which include aspects of 1) sales volume (business sales in the last three years have increased every year; increases on certain occasions (exhibitions/holidays)); 2) production results (total production continues to increase (in the last three years); the number of products that can meet market demand); 3) operating profit (business income increases every time there is a certain event (exhibition/holiday)); in general, operating income (in the last three years) has increased every year); 4) business growth (customers continue to increase every year (in the last three years)); customers come from various regions outside the current business area; product marketing is not only in the area where the business actors currently live); 5) business development (business actors: have more than one place of business; have opened a business branch outside the current place of business; have additional facilities (e.g., machines to support production processes/computers/laptops/notebooks/smartphones etc.) that support increased business production; adding types of businesses to support the operationalization of existing businesses to survive and maintain businesses during the Covid 19 pandemic).

### 3.3 Data analysis

The questionnaires and filling out questionnaires were distributed from August 2021 to March 2022 (7 months). The total questionnaires were distributed directly were 475 questionnaires (100% returned and can be used as research data), and questionnaires distributed online 150 times (66.67% or 100 respondents filled out the GF completely and sent it back), so the total respondents were 575 SME owners spread from these various regions.

### 3.4 Description of research respondents

Based on the results of distributing questionnaires to respondents, the characteristics of respondents are grouped by gender, age, regional origin, type of business, and length of business, which are presented in Tables 1- 3.

**Table 1:** Characteristics of respondents by gender and age.

Gender	Frequency	Percent (%)
Man	160	27.82
Woman	415	72.18
Age	Total	Percent (%)
20–25 years old	100	17.39
26–35 years old	149	25.91
35–45 years old	154	26.78
45–55 years old	125	21.75
>55 years old	47	8.17

In Table 1 and it is shown that the characteristics of respondents based on gender can be known to respondents of the male sex, namely 160 people (27.82%), and the number of female respondents, as many as 415 people (72.18%). The characteristics of respondents by age show that most respondents are in the age range of 35–45 years, namely 154 respondents or 26.78%, and at least age over 55 years, namely 47 respondents or 8.17%.

Table 2 describes the domicile of entrepreneurs and managers (business actors) who were respondents in this study. Business actors who were the respondents with the highest number domiciled in Magetan Regency, as many as 212 people or 36.87%; the second stayed in Madiun City, as many as 179 people or 31.13%; the third stayed in Sidoarjo as many as 85 people or 14.78%; the order of domiciled in Malang Regency as many as 27 people or 10.78%; the fifth place is domiciled in Ponorogo as many as three people or 0.53%, the sixth place is domiciled in Nganjuk and Jember City as many as two people or 0.35% each; and there are domiciled from the City of Surabaya, Blitar City, and Bondowoso as many as one people each or 0.17%. The largest group of business types was food and beverage businesses as many as 277 respondents or 48.17%; in the business group of daily product trade (convenience goods), as many as 151 respondents or 26.27%; in the furniture and building materials business group as many as 50 respondents or 8.70%; in the fashion group as many as 38 respondents or 6.60%, and in other groups including online shops, electronics, webbing, plant cultivation, farm) as many as 59 respondents or 10.26%. The highest length of business in the range of >5–10 years was 183 people or 31.83%; in the business duration of 1–5 years, as many as 172 people or 29.91%; in the long-range of business >10–15 years as much as 12%; in the long-range of >15–20 years of business as much as 6.96%; and business actors with a business length of 20 years > 18.43%.

**Tabel 2:** Characteristics of respondents based on domicile, type, and length of business.

Domicile	Total	Percent (%)
Magetan District	212	36.87
Madiun City	179	31.13
Sidoarjo Regency	85	14.78
Madiun Regency	62	10.78
Malang Regency	27	4.70
Ponorogo Regency	3	0.53
Nganjuk Regency	2	0.35
Jember City	2	0.35
Surabaya City	1	0.17
Blitar City	1	0.17
Bondowoso Regency	1	0.17
Business Type	Total	Percent (%)
Food and Beverage	277	48.17
Retailer (convenience goods)	151	26.27
Furniture and building materials	50	8.70
Fashion	38	6.60
Others (online shop, electronics, webbing, crop cultivation, farm)	59	10.26
Length of Business	Total	Percent (%)
<1 year	5	0.87
1–5 years	172	29.91
>5–10 years	183	31.83
>10–15 years	69	12.00
>15–20 years	40	6.96
>20 years	106	18.43

### 3.5 Validity test and reliability test

Construct assessment includes determining indicator reliability, internal consistency reliability, and convergent validity [97,98]. Internal consistency is evidenced by composite reliability and Cronbach alpha scores, all of which are expected to be above the value of 0.7 [98], and to assess convergent validity by measuring the average variance extracted (AVE) it is suggested that the AVE should be above 0.5 [99]. Assessment of the measurement model was carried out by looking at the standardized loading factor (SLF) value, the construct reliability (CR) value, the AVE value, and Cronbach's Alpha value (Table 3).

**Table 3:** Value loading factor dimensions measurement research variables.

Variables (Constructs)	Measurement Dimensions	SLF >0.5	AVE >0.5	CR ≥ 0.7	Cronbach's Alpha>0.70
ESE	Individual Beliefs (IB)	0.74	0.75	0.81	0.87
	Action (A)	0.73			
	Cognitive Resources (CR.)	0.69			
EL	Able to motivate (A.M.)	0.52	0.87	0.84	0.95
	Achievement Oriented (AO.)	0.81			
	Creative (C)	0.76			
	Flexible (F)	0.86			
	Persistent (P)	0.89			
	Risk-Taking (RT.)	0.69			
	Visionary (V)	0.66			
EM	Financial Reason (FR.)	0.59	0.67	0.71	0.82
	Social Reason (SCR)	0.58			
	Service Reason (SR.)	0.65			
	Self-fulfillment reason (SFR)	0.55			
EIB	Opportunity exploration (OE.)	0.66	0.56	0.74	0.92
	Ide Generation (IG.)	0.75			
	Championing (CH.)	0.78			
	Application (AP.)	0.79			
BP	Sales volume (SV.)	0.59	0.56	0.75	0.88
	Production result (PR.)	0.68			
	Operating profit (OP.)	0.70			
	Business growth (BG.)	0.76			
	Business development (BD.)	0.72			

Table 3 presents the results of the convergent validity test; it can be seen that the loading factor value of each indicator on the latent variable, with a loading factor value of> 0.50. SLF should be higher than 0.5 and, ideally, 0.7 or higher [100]. SLF values of 0.5 or 0.6 are still acceptable when CR, AVE, and CR are within the acceptable range. Table 3 shows AVE >0.50, and the indicator is considered valid. Likewise, the CR and Cronbach's Alpha values for each variable>0.70 indicate high internal consistency.

### 3.6 Structural equation analysis of SME business performance

#### 3.6.1 Conformity test of SME business performance structural equation model

After validity and reliability tests were carried out on all latent variables whose results were valid and reliable, a structural model conformity test was carried out on BP in SMEs in East Java. The complete business performance model is presented in a diagram as follows.

**Fig. 2:**
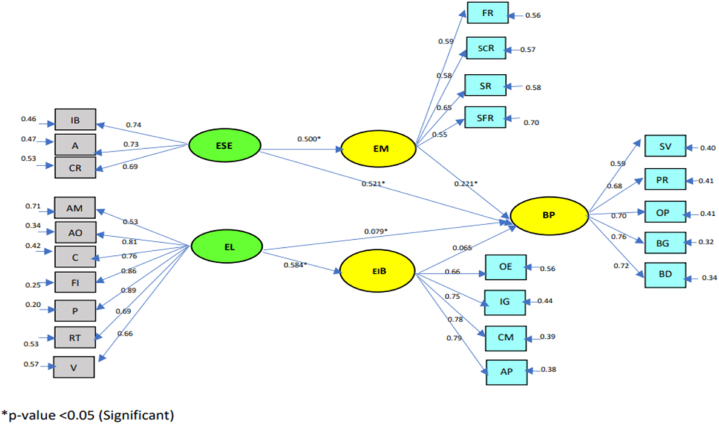
Relationship of exogenous variables to endogenous variables.

The test results of testing the relationship between exogenous and endogenous variables (Fig. 2) show one insignificant relationship in α = 0.05, namely the relationship between EIB and BP. Another test is by looking at the R^2^ value describing the GOF of a model, and the recommended R^2^ value is > 0. The results of this study data processing provide R-square values as shown in Table 4.

**Table 4:** GOF from R-square.

Model	R^2^
ESE →BP	0.272
EL → BP	0.079
ESE →EM	0.250
EL → EIB	0.341
ESE →EM →BP	0.309
EL → EIB → B.P	0.007

The value of R^2^ in Table 4 indicates that all values of R^2^ > 0. This shows that this research model already meets the criteria for Goodness of Fit. The result of calculating the value of Q squared based on Table 4 obtained the following results:

Q2 = 1- (1–0.272) x (1–0.079) x (1–0.250) x (1–0.341) x (1–0.309) x (1–0.007)) = 0.773.

It can be interpreted that the model can explain the overall business performance of 77.3%, and 22.7% is explained by other variables that are not studied. The results of the overall model match test can be seen in Table 5.

**Table 5:** *The goodness of fit Absolute Fit Measures* (GOF).

GOF size	Index	Value	Note
GFI	GFI >0.90 good fit; 0.80 ≤ GFI ≤0.90 marginal fit	0.89	Marginal fit
RMR	≤0.05	0.023	Good fit
RMSEA	≤0.08	0.066	Good fit
ECVI	Values that are small and close to ECVI saturated = 0.68	0.96	Marginal fit
NNFI	NNFI >0.90 good fit; 80 ≤ NNFI ≤0.90 marginal fit	0.94	Good fit
NFI	NFI >0.90 good fit; 0.80 ≤ NFI ≤0.90 marginal fit	0.93	Good fit
AGFI	AGFI >0.90 good fit; 0.80 ≤ AGFI ≤0.90 marginal fit	0.87	Marginal fit
RFI	RFI >0.90 good fit; 0.80 ≤ RFI ≤0.90 marginal fit	0.92	Good fit
IFI	IFI >.90 good fit; 0.80 ≤ IFI ≤0.90 marginal fit	0.95	Good fit
CFI	CFI >.90 good fit; 0.80 ≤ CFI ≤0.90 marginal fit	0.95	Good fit
PGFI	0.60–0.90	0.72	Good fit
PNFI	0.60–0.90	0.87	Good fit
CN	CN ≥ 200 good fit	201.18	Good fit

Hooper et al. (2008) assess the size of the model fit by looking at RMSEA, CFI, and RMR values. In Tables 5 and it can be seen that RMSEA, CFI, and RMR show a good fit; this means that it can be concluded that the model used in this study can be used as the basis for the analysis of research problems.

#### 3.6.2 Test the hypothesis of direct impact and indirect impact

The results of the regression analysis to be used in the direct influence hypothesis test are presented in Table 6.

**Table 6:** Summary of path analysis results.

Path	Coefficient of Influence	Standard Error	t-value	p-value	Conclusion
BP1 = γ_1_ESE + ς_1_ = 0.521ESE					
ESE→BP (path c)	0.521	0.039	14.625	0.000	Significant H1 accepted
EM = γ_1_ESE + ς_3_ = 0.500ESE					
ESE →EM (path a)	0.500	0.029	13.821	0.000	Significant H2 accepted
BP3 = γ_1_ESE + β1EM + ς_5_ = 0.411ESE+0.221EM					
ESE→ BP (path c’)	0.411	0.044	10.230	0.000	Significant
EM → BP (path b)	0.221	0.055	5.511	0.000	Significant H3 accepted
BP2 = γ _2_EL + ς_2_ = 0.079EL					
EL →BP (path c)	0.079	0.045	1.908	0.028	Significant H4 accepted
EIB = γ_2_EL+ ς_4_ = 0.584EL					
EL → EIB (path a)	0.584	0.033	17.201	0.000	Significant H5 accepted
BP4 = γ_2_EL+ β2EIB+ ς _6_ = 0.064EL+0.065EIB					
EL → BP (path c')	0.064	0.055	1.248	0.212	Insignificant
EIB→ BP (path b)	0.065	0.057	1.567	0.118	Insignificant H6 rejected

Table 6 illustrates that five hypotheses of direct influence are accepted, and one hypothesis (H6) is not accepted. H1, which states that ESE significantly improves BP, was accepted; this is indicated by the calculated t-value of 14.625< t of the table of 1.645 (one-sided test) with a p-value of 0.000 < 0.05. H2, which states that ESE significantly improves EM, is accepted; this is seen from t count 13.821 > 1.645 with a p-value of 0.000 < 0.05. H3, which states that EM significantly improves BP was accepted; the t count indicates this 5.511 < 1.645 with a p-value of 0.000 < 0.05. H4, which states that EL significantly improves BP, was accepted, evidenced by t count 1.908 > 1.645 with a p-value of 0.028 < 0.05. H5 states that EL improves EIB, which was accepted; this is indicated by t counting 17.201 > 1.645 with a p-value of 0.000 < 0.05. H6, which states that EIB positively improves BP, is not accepted; this is indicated by a calculated t-value of 1.567 < 1.645 with a p-value of 0.106 > 0.05.

H7 testing is ESE affects BP through EM as a mediator, and H8 testing affects EL on BP through EIB as a mediator using the Sobel test. The results of the mediation role test are presented succinctly in Table 7 below.

**Table 7:** Mediation variable influence test results.

Path	The test statistic (Sobel test)	Std. Error	p-value	Conclusion
ESE→ EM→ BP	3.913	0.282	0.000	H7 accepted
EL→ EIB→ BP	1.137	0.033	0.255	H8 rejected

Table 7 illustrates that H7, which states that ESE affects BP through EM as a mediator, was accepted. This is shown in the results of the Sobel test, showing that t count 3.913 > 1.645 with a p-value of 0.000 < 0.05. When referring to Ref. [101], the role of EM mediating the effect of ESE on BP is partially mediating because the impact of ESE on BP before and after the presence of EM in the regression equation decreases but remains significant (Table 6; Tabel 7).

The 8th hypothesis test, which states that EL affects BP through EIB as a mediator, was not proven. This is shown in the results of the Sobel test, showing that the t count is 1.137 < 1.645 with a p-value of 0.255 > 0.05. Referring to Ref. [102] about the requirements for mediation variables, it can be seen that one of the conditions for the mediating variables in the EIB is not met; namely, the effect of EIB on BP is insignificant (Table 6), so it can be concluded that EIB does not mediate the impact of EL on BP in SMEs in East Java.

## 4 Discussion

### 4.1 The impact of ESE on BP

ESE is an entrepreneur's belief in self-competence in facing tasks, difficulties, and uncertainties in running his business under certain conditions, for example, during the Covid-19 pandemic. Successful individuals have higher SE than individuals who fail in their efforts. ESE can be seen as a predictor factor over the high performance that a person must show in the entrepreneurial process. The empirical testing of 575 SMEs revealed that ESE significantly increased BP. ESE is the dominant predictor compared to other exogenous variables included in the model*.* The results of previous empirical studies also prove that when the ESE is high, it can improve BP [52,75]. The results of other empirical studies that support [103] show that ESE is a component of individual personality that contributes significantly positively to the performance of early-stage startup companies.

These results support the SCT and TPB. ESE states that the level of control of the individual's behavior is the primary determinant of a person's intention to engage in certain behaviors [104], such as being persistent in learning new things and daring to take risks. With the learning process of managing a business, an entrepreneur can develop knowledge, skills, positive behaviors, and the power of innovating in business processes effectively and efficiently so that entrepreneurs can improve BP.

### 4.2 The effect of ESE on EM

The study results revealed that ESE significantly increased the EM of SMEs in East Java. These results follow TPB that perceptions or behavior of the level of difficulty or ease of a thing affect individual motivation. That is, if a person has high self-efficacy to start a business, he will have a sense of optimism to start a business, so the person's EM will increase. SE is significantly positively related to motivation [105]. The study's results reinforce previous empirical studies [39,106], which prove that the higher the ESE, the higher the EM. Confidence in one's abilities can give rise to the need for self-fulfillment and self-progress in addition to meeting other conditions such as financial, social, and service to other parties. This proves that ESE can influence self-motivation as an individual factor; for example, explicit and challenging goals can maintain and improve one's motivation.

## 5 The effect of EM on BP

A person who has a strong EM means that someone already has the drive and desire from within himself to carry out the role of entrepreneurship with the passion for achieving BP. This follows the TPB; motivation is associated with subjective norms. Subjective norms are a person's views on things that can affect a person's intention or interest in doing an action, while motivation is the impulse to act. This means that motivation can affect a person's interest in acting. The higher a person's motivation in entrepreneurship, the higher one's success in entrepreneurship. The results of empirical studies show that EM as measured by financial reason, social reason, service reason, and self-fulfillment reason, can significantly increase BP. The results of previous empirical studies [80] proved a significant positive impact of motivational factors on the survival success of a business. Likewise, the results of other studies [39,59,107] prove the significant positive impact of EM on business success.

### 5.1 The effect of EL on BP

Any leader in an organization can influence a company's performance; better leadership practices impact the company's performance. EL is important in improving BP [49]. Business owners must have leadership and entrepreneurial skills to run a successful business. The results of this empirical study show that EL has a significant positive effect on the BP of SME owners in East Java. This indicates that the higher EL business owners apply to manage their businesses will improve BP. The results of empirical studies from this study support several previous empirical studies [105,108] that showed a positive and significant effect of EL on BP. Entrepreneurial leaders are leaders who have an entrepreneurial spirit, which is different from leaders in general because they have characteristics such as being able to motivate themselves and others, having an orientation to high achievement, being creative, having high flexibility over environmental dynamics, being persistent, being able to recognize and read market opportunities, being able to take into account, consider, and take risks on previously recognized opportunities, and able to allocate the resources it has effectively and efficiently. These characters are predicted to improve the performance of their business.

### 5.2 The effect of EL on EIB

EL is an innovative entrepreneur who experiments aggressively and skillfully practices the transformation of opportunities attractively. The results of this empirical study follow the results of previous empirical studies [43,44], which show a significant positive influence of EL on EIB; while [7] show that EL has a positive effect on EIB. The results showed that entrepreneurial leaders, with their innovative behaviors, could deliberately influence employees' innovative behavior and provide an innovative business environment to generate creative and new ideas and realize them without fear of failure or error. Another study by Ref. [108] on women entrepreneurs in the solar energy sector from Lima, Peru, showed that EIB is influenced by entrepreneurial coaching. The results of another study on women entrepreneurs in Nigeria showed that self-efficacy and internal locus of control positively and significantly influenced EIB in these women entrepreneurs [109].

Innovative entrepreneurial leaders can influence employees' innovative behavior through conscious and unconscious actions with the ownership of goals that can stimulate the generation of ideas and their applications. EL creates a promising environment by supporting its subordinates to abandon traditional ways of thinking and move on to new methods, create new ideas, and provide innovative solutions to problems faced by business leaders [7]*.* When the business environment is dynamic and competitive, leaders play a strategic role in business sustainability, business performance, and business growth through innovation processes [76]. Improving EL is a strategic effort for business leaders to maximize the EIB of business leaders in running their current business.

### 5.3 The effect of EIB on BP

EIB is a comprehensive policy for improving business competence. EIB can be interpreted as the production of a useable product, process, or service that comes from the identification of problems and the generation of ideas EIB can also be interpreted as the creation of an introduction, the application of new ideas in work to improve the performance of the roles of individuals, groups, and organizations. The results of hypothesis testing showed that EIB did not significantly affect business performance. Nevertheless, when viewed from the direction of the relationship, it is known that the influence is positive (Table 6). This result indicates that the EIB owned by the SME owner has not been able to improve the current business performance significantly. These results are inconsistent with some of the results of previous empirical studies [42,89], which showed a positive and significant influence of IB on business performance.

The results of this empirical study, which do not correspond to previous studies, are suspect that most of the East Java SME owners who are respondents to the study have been unable to maximize EIB. The non-optimal EIB is indicated by the length of business of most respondents in the range of >5–10 years (31.83%) and 1–5 years (29.91%). In line with the opinion of [49] that IB determines innovative capabilities, which in the end can have a positive impact on BP, then be able to increase the influence of EIB on the performance of SME.

### 5.4 The effect of ESE on BP through EM as mediating

In supporting the improvement and development of SMEs, ESE is needed, which is essential in increasing EM in achieving a job's success, which is associated with BP. ESE is also a driving factor that makes a person more active and optimal in working. ESE can affect BP through attitudes and behaviors through actions that are oriented towards achieving achievements or performance by utilizing their abilities. EM is an entrepreneurial effort to create and explore business opportunities in society regarding social, environmental, and economic opportunities [110]. Similarly, a strong EM will impact BP or, in this case, the performance of SMEs. Business actors with high self-efficacy feel confident in their abilities and always feel optimistic about running their business, so strong motivation in entrepreneurship will arise to improve business performance. The results of this study are similar [28], which shows that EM mediates the influence of ESE on business success. Another similar study [111] also showed that motivation plays a role in mediating the influence of SE on entrepreneurial achievements.

### 5.5 The effect of EL on BP through EIB as mediating

EL has a heavy point on concepts and ideas related to problems associated with individual behavior, such as decision-makers, problem solvers, risk-makers, strategic initiatives, and vision determinants. EL is essential to raise the performance of firms [49]. EL is seen as effectively capable of increasing competitiveness in overcoming an uncertain environment and achieving sustainable organizational development [112], so this leadership model fosters innovative behaviors. From the results of testing the hypothesis of the role of EIB mediation, it is shown that EIB does not mediate the influence of EL on BP on SMEs in East Java. The results of this study have not been able to provide empirical evidence supporting [49] that innovative behavior can ultimately positively impact business performance. Meanwhile, a study [93] revealed that the effect of EL on BP is fully mediated by team creativity.

## 6 Conclusions and their implications

From the results of hypothesis testing, it can be concluded as follows: 1) ESE affects positively and significantly BP; 2) ESE affects positively and significantly EM; 3) EM positively and significantly affects BP; 4) EL positively and significantly affects BP; 5) EL positively and significantly affects EIB; 6) EIB does not significantly affect BP; 7) EM mediates the influence of ESE on BP partially; 8) EIB does not mediate the influence of EL on BP. The contribution of this study is to provide a basic conceptual framework that can be useful in driving SME performance through a study of the internal factors of business actors that can influence SME development, namely self-efficacy, motivation, leadership, and innovative behavior from business actors. The results of this study support the RBV, which focuses on internal factors, which are the fundamental variables of the company and its performance, especially from the aspect of intangible resources such as knowledge or motivation and abilities. In a rapidly changing business environment, these four internal factors are essential to generate competitive advantage and improve SME performance.

## Implications for public policy and entrepreneurs

ESE is a dominant predictor of BP in SMEs in East Java, so there needs to be a strong emphasis on business actors in this aspect so that a high ESE for SME owners is expected to increase the sense of self-efficacy and work motivation in the work team or its employees. Therefore, novice entrepreneurs are advised to participate in ESE development workshops to increase BP. ESE also improves EM, and EM also improves business performance. EM is the second-order predictor for BP in SMEs in East Java, so EM entrepreneurs need to continue to improve because, with a strong motivation in running their business, it will be able to provide a strong encouragement for employees so that employees are also eager to help SME owners achieve the business goals set (business success). These results indicate that entrepreneurship, besides requiring high ESE, also requires encouragement, enthusiasm, interest, and high motivation for someone to want to achieve business success. This research contributes to the entrepreneurial behavior literature, which has implications for policymakers and educators in developing an entrepreneurial spirit for young people who want to start a career in entrepreneurship.

EL can increase EIB and BP. This indicates that EL has a strategic role in increasing innovative behavior and business breakthroughs so that SMEs can maintain business continuity in the long term. Entrepreneurial leadership will have a positive or negative impact depending on the leader's ability to make decisions, see opportunities, and manage all the resources SMEs own. One of the efforts that stakeholders can make in improving the performance of SMEs is increasing EL through training according to the needs and demands of the business environment, which can be carried out periodically.

EIB has no significant impact on business performance. Therefore, business actors must be improved again to foster innovative behavior in work teams. Increasing innovation behavior can be done by utilizing existing social capital in the SME network that is followed. This must be done because business actors face a dynamic business environment, thus requiring optimal business creativity and innovation for continuous improvement and development. The government needs to increase its intervention in the digitalization process for SMEs and be more proactive in supporting innovative business activities so that SMEs are ready to contribute actively in the era of society 5.0. SME owners, especially novice entrepreneurs, are expected to be proactive in participating in entrepreneurship coaching to enhance their ability to innovate in entrepreneurship to improve the performance of the businesses they start.

## Limitations and implications for research

The research sample was obtained based on the uncontrolled quota sampling technique, which is a sampling technique that is always carried out by not imposing any restrictions on the choice of researcher samples because the population of SMEs in East Java is substantial and continues to grow. There is no adequate SME database in every city and district in the East Java region. Moreover, the results of this empirical study have not been able to prove the significant effect of EIB on BP, so for the development of further research, it can be retested using similar research objects (SMEs) but focusing on the creative industry. Testing the influence of EL on BP can use EM as a mediating. In the development of further research, proactive personality can be used as a mediating variable for the impact of EL on EIB. Another exploration of the determinants of BP can consider external factors of entrepreneurship, such as digital marketing, human technology, and financial technology. For further research, improving the quality of variable measurements is recommended. For example, ESE measurement can be developed into 5 dimensions to measure ESE concerning business opportunity discovery, business planning, resource collection, placing people, and the use of financial resources [53]. This study did not include age, gender, level of education, or how long the company has been established as control variables so that future research can use these variables as control variables. Future researchers can investigate these variables by adding variables (eg, social capital, technological innovation capabilities) to understand better the factors that influence SME performance. Further research can be focused on young entrepreneurs, considering the results of a study [58] that revealed a significant correlation between their survival and opportunity motivation and their subjective and social psychology.

## Data availability statement

Data included in article/supp. material/referenced in article.

## Additional information

No additional information is available for this paper.

## CRediT authorship contribution statement

**Veronika Agustini Srimulyani:** Conceptualization, Data curation, Investigation, Methodology, Software, Writing – original draft. **Yustinus Budi Hermanto:** Conceptualization, Funding acquisition, Resources, Supervision, Validation, Visualization, Writing – review & editing. **Sri Rustiyaningsih:** Data curation, Formal analysis, Investigation, Methodology. **Laurensius Anang Setiyo Waloyo:** Data curation, Formal analysis, Methodology, Project administration.

## Declaration of competing interest

The authors declare that they have no known competing financial interests or personal relationships that could have appeared to influence the work reported in this paper.
